# Mortality Predictors and Retrospective Concordance with ICU Admission Prioritization Guidelines in Patients with Cancer: A Single-Center Cohort Study

**DOI:** 10.3390/cancers18183056

**Published:** 2026-09-21

**Authors:** Katarzyna Kosz, Justyna Wasiewicz, Amelia Bień, Maksymilian Skwirut, Paweł Piwowarczyk, Michał Borys

**Affiliations:** 1 Faculty of Medicine, The John Paul II Catholic University of Lublin, 20-708 Lublin, Poland; 2 Anesthesiology and Intensive Care Department, Center of Oncology of the Lublin Region, 20-090 Lublin, Poland; 3 Faculty of Medicine, Medical University of Lublin, 20-059 Lublin, Poland

**Keywords:** cancer, intensive care, mortality, Acute Physiology and Chronic Health Evaluation II (APACHE II), Eastern Cooperative Oncology Group (ECOG) performance status, triage, retrospective cohort

## Abstract

Background/Objectives: Advances in cancer treatment and an aging population have increased ICU admissions among patients with cancer, while mortality remains high and ICU resources are limited. We aimed to identify mortality predictors available at admission and factors arising during the ICU stay and to evaluate retrospective concordance with ICU admission-prioritization guidelines. Methods: This retrospective single-center cohort included 287 patients with an active malignancy admitted to an oncology ICU in Lublin, Poland, from 1 January 2024 through 31 December 2025. Two independent reviewers, blinded to outcomes, retrospectively assigned ICU admission priorities according to Polish Society of Anaesthesiology and Intensive Therapy guidelines. Univariable and multivariable logistic regression analyses were used to identify factors associated with ICU mortality. Results: Overall ICU mortality was 41.1% (118/287). In the adjusted admission-time model, APACHE II score (odds ratio (OR), 1.15 per point), ECOG 4 (OR, 6.37), and postoperative admission (OR, 0.29) were associated with mortality (area under the receiver operating characteristic curve (AUC), 0.84). In the exploratory ICU-course model, mechanical ventilation >48 h or death within 48 h while mechanically ventilated (OR, 6.79) and vasopressor therapy >48 h or death within 48 h while receiving vasopressors (OR, 4.33) were associated with mortality. The proportion of priority-4 admissions decreased from 19.2% (24/125) in 2024 to 4.9% (8/162) in 2025 (*p* < 0.001). Priority-4 classification remained associated with ICU mortality even after adjustment for APACHE II score. Conclusions: Cancer, even at an advanced stage, should not be regarded as an absolute contraindication to intensive care. Clinical and oncological information available at admission may support evidence-based ICU triage. Structured prioritization may help identify patients with a very limited likelihood of benefiting from intensive care and support responsible allocation of critical care resources.

## Simple Summary

Some patients with cancer require treatment in an intensive care unit (ICU), but it can be difficult to identify who is most likely to benefit. We reviewed 287 adults with an active malignancy who were admitted to a specialist oncology ICU in Poland during 2024–2025. Forty-one percent died in the ICU. Greater illness severity at admission, mechanical ventilation and vasopressor therapy were associated with a higher risk of death, whereas postoperative admission was associated with a lower risk. Retrospectively assigned priority 4 was strongly associated with ICU mortality. These findings suggest that structured admission criteria may support consistent ICU triage, but they do not prove that greater concordance with the guidelines caused better survival. Confirmation in prospective multicenter studies is needed.

## 1. Introduction

Outcomes among patients with cancer have improved substantially during the past decade because of advances in systemic therapy, radiotherapy, minimally invasive surgery, and individualized perioperative care [1,2,3]. Nevertheless, some patients require intensive care unit (ICU) admission for sepsis, postoperative monitoring, acute respiratory failure, cardiovascular or neurologic complications, or acute kidney injury [4].

Survival among critically ill patients with cancer has improved since the beginning of the century [5]. Several factors may explain this improvement. Cancer screening and diagnostic modalities have become more effective and accessible [6,7]. Healthcare professionals are also increasingly aware of malnutrition and nutritional requirements [8], which are especially relevant in patients with cancer because tumor-associated metabolic changes can promote cachexia [9]. Nutritional screening is recommended for patients with cancer and for new hospital admissions more generally [10], while enteral and parenteral formulations, as well as oral nutritional supplements, can support individualized nutritional care [11,12]. Robotic and minimally invasive surgery may reduce complications and hospital length of stay [13] and can expand treatment options for older and frailer patients [14]. Finally, newer immunotherapies have altered both the efficacy and toxicity profiles of systemic anticancer treatment [15]. Collectively, these developments have changed the population of patients with cancer who may require or benefit from intensive care.

A recent scoping review reported an average ICU mortality of approximately 41%, although estimates ranged from 8% to 72% because of substantial between-study heterogeneity [16]. Patients with cancer are often analyzed as a subgroup of a general ICU population [17,18,19], whereas evidence from dedicated oncology ICUs remains limited [20]. At our Cancer Center, approximately 90% of ICU patients have an oncological diagnosis.

Admission of patients with advanced or palliative-stage disease raises additional clinical and ethical challenges. The Society of Critical Care Medicine describes prioritization, diagnosis, and objective-parameter models for ICU admission [21]. The Polish Society of Anaesthesiology and Intensive Therapy adopted a four-level prioritization model in national guidance published in 2022 [22]. The extent of retrospective concordance between ICU admissions and this guidance has not been well characterized.

The primary objective of this study was to identify factors associated with ICU mortality among patients treated in a specialist oncology ICU. Secondary objectives were to assess retrospective concordance with national ICU admission-prioritization guidance and to compare priority-4 admissions between 2024 and 2025. We hypothesized that admission priority would be associated with ICU mortality.

## 2. Materials and Methods

### 2.1. Study Design and Setting

We conducted a retrospective single-center cohort study at the Department of Anesthesiology and Intensive Care Medicine, Center of Oncology of the Lublin Region. The study period extended from 1 January 2024 through 31 December 2025. Reporting followed the Strengthening the Reporting of Observational Studies in Epidemiology (STROBE) statement. The study protocol was approved by the Bioethics Committee of the John Paul II Catholic University of Lublin (approval no. 17/2026; 30 June 2026).

### 2.2. Participants

**Figure 1:**
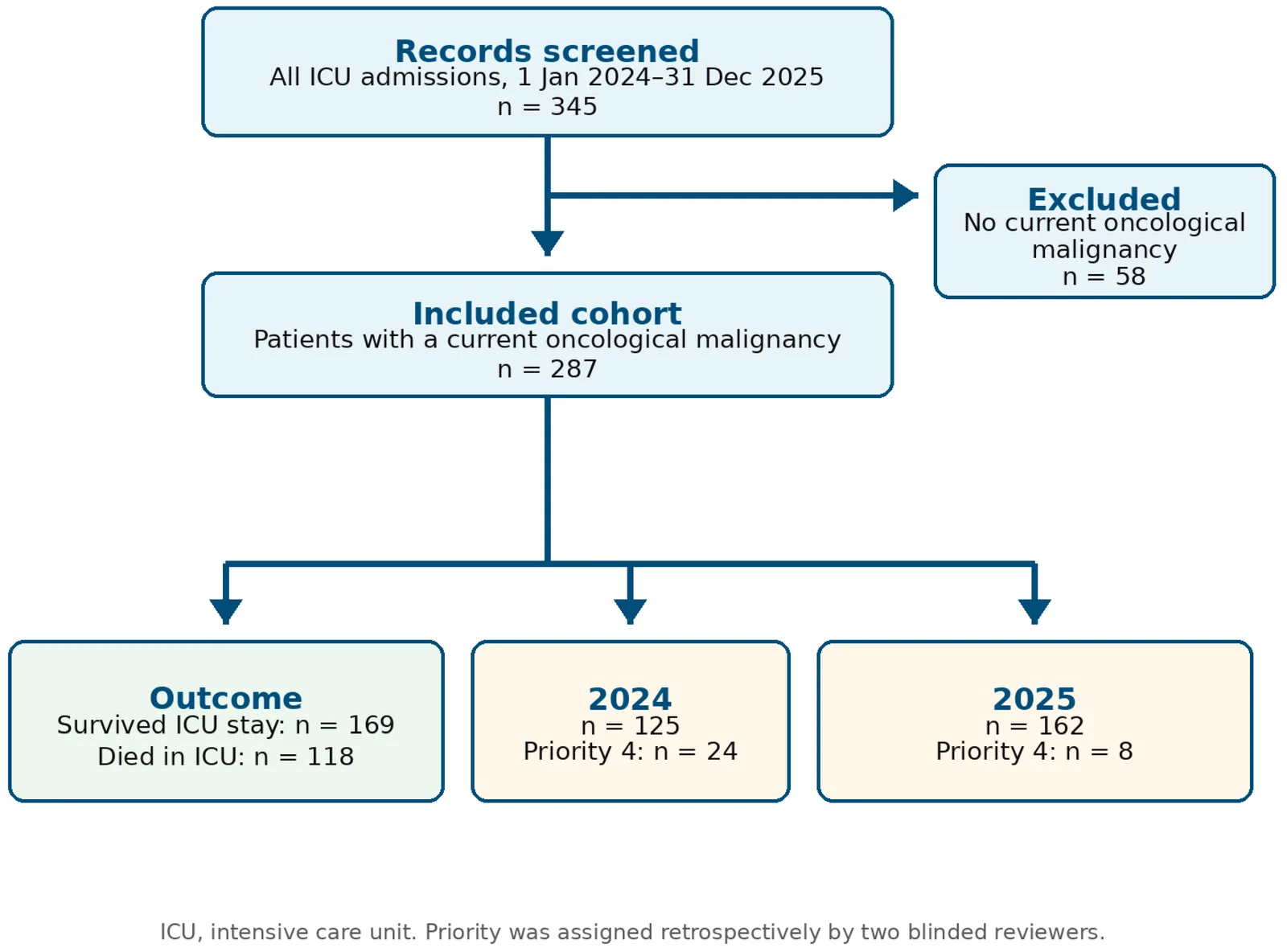
STROBE flow diagram of participant selection and analysis.

All patients admitted to the ICU during the study period were screened. Patients were eligible if they had a current oncological malignancy. Of 345 screened patients, 58 were excluded because they did not have a current malignancy, leaving 287 patients in the analytic cohort (Figure 1).

### 2.3. Variables and Data Sources

Variables extracted retrospectively from medical records and available at ICU admission included age, sex, Acute Physiology and Chronic Health Evaluation II (APACHE II) score, Eastern Cooperative Oncology Group (ECOG) performance status at admission, admission type (surgical or non-surgical), malignancy type (solid or hematologic), solid-tumor stage and metastatic status, and recent anticancer treatment including radiotherapy and chemotherapy. Solid tumors were classified using the TNM system [23]. Variables describing the subsequent ICU course included ICU length of stay, mechanical ventilation lasting >48 h or death before 48 h, vasopressor therapy lasting >48 h or death before 48 h, and microbiological culture results obtained during the ICU stay. For the exploratory ICU-course analysis, mechanical ventilation and vasopressor therapy were analyzed as composite variables. Patients were classified as exposed if they received the respective organ support for >48 h or if they died within the first 48 h while receiving that support. These composite variables were intended to characterize an adverse ICU course requiring ongoing organ support or early death during organ support and were not considered measures of treatment duration alone. The temporal benchmark for classification was availability at the time of ICU admission: variables available at or around ICU admission and potentially available for the initial clinical assessment were classified as admission-time characteristics, whereas events, treatments, or results arising during the subsequent ICU stay were classified as post-admission variables. Post-admission variables were therefore unavailable for initial ICU triage and were analyzed separately.

### 2.4. Retrospective Assessment of Admission Priority

Two independent reviewers retrospectively assessed whether each ICU admission was consistent with the 2022 Polish Society of Anaesthesiology and Intensive Therapy guidance [22], which is based on Society of Critical Care Medicine guidance [21]. Priority 1 indicates the strongest recommendation for immediate ICU transfer, whereas priority 4 identifies patients considered unlikely to benefit from ICU admission. Priority classification was based exclusively on information available at ICU admission; reviewers had sufficient information to assign priority to all 287 patients and were blinded to the subsequent treatment course and clinical outcome. Disagreements were adjudicated by a third researcher. The classification was performed retrospectively and was not used to guide actual ICU admission decisions. Therefore, the analysis assessed retrospective concordance with guideline-based prioritization rather than prospective adherence to or implementation of the guidance. Pre-consensus interobserver agreement was assessed using exact percentage agreement and weighted Cohen’s kappa.

### 2.5. Outcomes

The primary outcome was all-cause ICU mortality. Secondary outcomes were the distribution of admission-priority categories, the proportion of priority-4 admissions by calendar year, and associations between candidate clinical variables and ICU mortality.

### 2.6. Statistical Analysis

Continuous-variable distributions were evaluated using the Shapiro–Wilk test. Continuous variables were non-normally distributed and were summarized as medians with interquartile ranges (IQRs); groups were compared using the Mann–Whitney U test. Categorical variables were summarized as counts and percentages and compared using the chi-square test or an exact test when expected cell counts were insufficient for asymptotic inference. Because of the sparse priority 3 category, the overall comparison of the four admission-priority categories according to ICU survival was performed using an exact test for the 4 × 2 contingency table (Fisher–Freeman–Halton exact test). Associations with ICU mortality were evaluated using univariable and multivariable logistic regression separately for variables available at ICU admission and variables arising during the subsequent ICU stay. The principal admission-time multivariable model included variables selected a priori to represent clinically distinct domains available at or around ICU admission: age, APACHE II score, ECOG performance status, admission context (surgical/postoperative vs. non-surgical), and malignancy type (hematological vs. solid). Post-admission variables were not included in this model.

The association between priority-4 classification and ICU mortality was analyzed separately from the principal admission-time model because it represents a composite guideline-based clinical classification integrating several aspects of clinical status and anticipated benefit from intensive care, rather than an individual clinical characteristic. Priority 4 was compared with priorities 1–3 in univariable logistic regression as the reference category. Given the relationship between priority-4 classification and acute physiological severity, an additional model evaluated this association after adjustment for APACHE II score. Priority was analyzed separately from the principal admission-time model because it represents a composite clinical classification rather than an individual clinical characteristic. Reference categories were priorities 1–3 for priority 4, solid malignancy for hematologic malignancy, ECOG 1 for ECOG 4, non-surgical admission for surgery, and absence of the respective exposure for binary variables. Associations with ICU mortality are reported as odds ratios (ORs) with 95% confidence intervals (CIs). Discrimination of the admission-time model was assessed using the area under the receiver operating characteristic curve (AUC). The sampling variability of the AUC was estimated using 1000 nonparametric bootstrap resamples; in each replication, a sample of the same size as the original analytical sample was drawn with replacement, the model was refitted, and the AUC was recalculated. The standard deviation of the bootstrap distribution of AUC estimates was used as the bootstrap standard error. This procedure was used to quantify the precision of the apparent AUC and was not intended as optimism-corrected internal validation.

A separate exploratory multivariable logistic regression analysis evaluated factors occurring during the ICU stay, including mechanical ventilation >48 h or death within 48 h while mechanically ventilated, vasopressor therapy >48 h or death within 48 h while receiving vasopressors and positive blood cultures. These composite organ-support variables and positive blood cultures were evaluated in a separate exploratory multivariable model and were not included in the principal admission-time model. Because the organ-support variables incorporate events occurring after ICU admission, including early death while receiving support, this analysis was considered descriptive and exploratory rather than an admission-time prognostic model. Accordingly, this analysis was interpreted as showing exploratory associations with ICU mortality rather than admission-time prognostic effects. Model discrimination was assessed using the AUC with 95% CIs. Analyses were performed using Statistica version 13.1 (TIBCO Software Inc., Palo Alto, CA, USA) and Stata version 18.5 (StataCorp LLC, College Station, TX, USA). All tests were two-sided, and a *p*-value < 0.05 was considered statistically significant.

## 3. Results

### 3.1. Participants and Characteristics

The analysis was conducted in July and August 2026. Of 345 screened ICU patients, 287 had a current malignancy and were included (Figure 1). Overall, 118 patients (41.1%) died in the ICU and 169 (58.9%) survived to ICU discharge. Patient characteristics and unadjusted comparisons by ICU survival status are shown in Table 1.

### 3.2. Admission Priority, Mortality, and Interobserver Agreement

The proportion of patients retrospectively classified as priority 4 was lower in 2025 than in 2024: 24 of 125 (19.2%) versus 8 of 162 (4.9%), respectively (*p* < 0.001). Priority-4 classification was strongly associated with ICU mortality. Mortality was 84.4% (27/32) among patients classified as priority 4 compared with 35.7% (91/255) among those classified as priorities 1–3. Compared with patients classified as Priorities 1–3 combined, patients classified as priority 4 had substantially higher odds of ICU mortality (OR 9.73, 95% CI 3.62–26.14; *p* < 0.001). After adjustment for APACHE II score, priority 4 remained associated with ICU mortality (adjusted OR 4.96, 95% CI 1.56–15.80; *p* = 0.007), while APACHE II score remained associated with mortality (OR 1.15 per point, 95% CI 1.10–1.20; *p* < 0.001). Pre-consensus interobserver agreement for admission-priority classification was high. The two independent reviewers agreed in 273 of 287 cases (95.1%), with a weighted Cohen’s kappa of 0.93 (*p* < 0.001). All disagreements involved adjacent priority categories.

### 3.3. Logistic Regression

Univariable logistic regression results are shown in Table 2. Higher APACHE II score, priority-4 admission, hematological malignancy, ECOG 4, prolonged organ support, metastatic disease, recent radiotherapy or systemic therapy, and positive cultures were associated with higher odds of ICU mortality. Surgery was associated with lower odds of ICU mortality.

In the admission-time multivariable analysis, higher APACHE II score (OR 1.15 per point, 95% CI 1.10–1.21; *p* < 0.001) and ECOG 4 compared with ECOG 1 (OR 6.37, 95% CI 2.05–19.78; *p* = 0.001) were associated with higher ICU mortality (Table 3). Surgical/postoperative admission was associated with lower ICU mortality (OR 0.29, 95% CI 0.14–0.60; *p* = 0.001). Age, ECOG 2, ECOG 3, and hematological versus solid malignancy were not significantly associated with mortality after adjustment. The admission-time model had an AUC of 0.84 (95% CI 0.79–0.88). In 1000 nonparametric bootstrap resamples, the bootstrap standard error of the AUC was 0.024, with a similar 95% CI (0.79–0.88). The Brier score was 0.159. These performance measures describe the model in the development cohort and should not be interpreted as optimism-corrected internal validation.

In the separate exploratory analysis restricted to ICU-course variables, the composite mechanical ventilation and vasopressor therapy variables were independently associated with ICU mortality (Table 4), whereas positive blood culture did not reach statistical significance. These associations represent exploratory ICU-course findings and should not be interpreted as admission-time prognostic effects.

## 4. Discussion

In this retrospective cohort from a specialist oncology ICU, 41.1% of patients died before ICU discharge. Greater acute illness severity and poorer functional status at admission were independently associated with mortality, whereas postoperative admission was associated with lower mortality. Patients with hematologic malignancies had worse unadjusted outcomes than those with solid tumors. Retrospectively assigned priority 4 was strongly associated with mortality, and the proportion of priority-4 admissions was lower in 2025 than in 2024. Our analysis extends the available evidence by separating admission-time predictors from ICU-course variables and by evaluating admission-priority classification independently. This distinction avoids conflating information available during triage with events that occurred after admission. These findings also illustrate why prognostic models and triage frameworks should be interpreted at different levels. APACHE II and ECOG describe acute severity and baseline function, whereas admission priority integrates clinical judgment and the anticipated benefit of organ support. Their associations with mortality do not establish a threshold beyond which treatment is non-beneficial. Instead, they may structure multidisciplinary discussion, identify cases requiring reassessment, and support transparent documentation of decisions.

The reduction in priority-4 admissions from 2024 to 2025 suggests greater concordance with national prioritization guidance, but the before–after comparison cannot establish causality. Secular changes in referral patterns, staffing, education, bed availability, case mix, or documentation may have contributed. Furthermore, priority was reconstructed retrospectively rather than assigned during real-time triage. Accordingly, the results support further evaluation of structured prioritization; they do not support denial of ICU care solely on the basis of a priority category. The high pre-consensus agreement indicates that the criteria could be applied reproducibly by reviewers using documented admission data. However, retrospective agreement does not demonstrate bedside feasibility. In prospective practice, incomplete information, time pressure, goals-of-care discussions, and an evolving clinical response may create greater uncertainty. Future evaluations should therefore assess not only interobserver agreement but also timeliness, treatment decisions, resource use, survival, quality of life, and concordance with patient preferences. This distinction is clinically important because population-based data show high in-hospital mortality and short post-discharge survival after in-hospital cardiopulmonary resuscitation among patients with stage IV cancer [24]. When limitation of life-sustaining treatment is considered, recent ESICM guidance supports a structured multidisciplinary decision-making process that incorporates the patient’s preferences and values, effective communication with relatives, and early integration of palliative care [25].

In some clinical scenarios, determining whether ICU admission and life-sustaining therapy are beneficial is difficult, especially when the patient’s prognosis remains uncertain. Thus, over the last decade, a time-limited trial (TLT) has become a useful approach [26]. It involves initiating intensive treatment, either without restrictions or with previously agreed-upon limits, for a predefined period. In 2024, the American Thoracic Society Workshop Committee established a consensus regarding TLT [27]. The aim of this therapy is to observe the patient’s clinical response and obtain additional information about the likelihood of reversibility of the ongoing process. Furthermore, TLT gives the patient and family members time for better comprehension of the functional limitations of a human being during cancer treatment. It also explores the ethical issues that may arise during TLT.

The observed ICU mortality in our study was consistent with the pooled average reported in a recent scoping review of 73 studies involving more than 170,000 ICU admissions and with several individual cohorts [16,28,29,30,31,32,33]. Differences from the lower 90-day mortality reported after unplanned ICU admission in a multicenter study may reflect case mix, admission type, outcome definition, and local organizational factors [20]. The lower mortality observed among postoperative admissions in our cohort is also directionally consistent with findings of better short- and medium-term survival among patients with solid cancer admitted to surgical rather than medical ICUs [19].

Patients with hematologic malignancies had higher unadjusted mortality, consistent with some previous reports [34,35]. Severe complications after stem-cell transplantation or cellular therapy and invasive fungal infection may contribute to risk [36]. In the recent systematic review by Quintana on hematological patients admitted to the ICU, the authors analyzed 25 studies from multiple continents [37]. Among all studies included in the review, only three were prospective. They presented a very vast range of ICU mortality, from 24.8% in the U.S study to 84.1% in the South Korean report [38,39]. The authors of the review identified common predictors of mortality across most studies, including invasive mechanical ventilation, vasopressor use, and sepsis. These predictors are similar to those shown in our study. In a retrospective analysis of five university hospitals in the Netherlands, the authors reported a 38% 1-year survival rate in their multicenter study involving over 1000 hemato-oncological patients [40]. They found a strong association between multiorgan failure and the final outcome, with 8% survival in patients with four-organ failure.

APACHE II and ECOG performance status were associated with mortality, broadly consistent with previous studies [28,29,30,41,42]. Age was not associated with mortality in this cohort. Metastatic disease, however, was associated with higher unadjusted mortality, consistent with prior reports [30,43]. Recent radiotherapy and systemic anticancer treatment were also associated with mortality in unadjusted analyses; these findings may reflect confounding by cancer severity and treatment indication rather than harmful effects of treatment itself.

This study has several limitations. Its retrospective, single-center design limits causal inference and generalizability. Admission priority was reconstructed from documentation and may not reproduce real-time decisions. Therefore, the study assesses retrospective concordance with guideline-based prioritization rather than prospective adherence. The small number of patients in some priority categories, particularly priority 3, limited category-specific statistical inference. We did not perform optimism-corrected internal validation or external validation. The analysis focused on ICU mortality and did not assess hospital, 30-day, or 90-day mortality; return to anticancer therapy; or patient-centered outcomes. Residual confounding is likely because nutritional status, sarcopenia, inflammatory markers, and comorbidity indices were unavailable. The organ-support variables used in the exploratory ICU-course analysis were composite measures defined as support lasting >48 h or death within 48 h while receiving the respective support. Their classification therefore partly depends on subsequent clinical course and outcome, which may introduce outcome-dependent classification and precludes interpretation as duration-based prognostic effects. A time-dependent analytical approach would be preferable for specifically evaluating associations between duration of organ support and mortality. Accordingly, these findings should be regarded as exploratory. The observed temporal change in priority-4 admissions cannot be attributed solely to guideline implementation. The observed associations between treatment modalities, such as radiotherapy and chemotherapy, and mortality in unadjusted regression analyses may reflect confounding by indication or by underlying disease severity. Prospective multicenter validation is required.

## 5. Conclusions

In this single-center cohort of patients with active malignancy, ICU mortality was associated with acute illness severity at admission and with clinical events arising during the ICU stay. Retrospectively assigned priority 4 identified patients with poor outcomes, but it should not be used as a stand-alone rule for individual treatment limitation. Structured prioritization may support transparent, multidisciplinary decision-making and responsible resource use; its clinical impact should be tested prospectively using patient-centered outcomes and methods that account for time-dependent exposures.

## Figures and Tables

**Table 1:** Characteristics of the cohort according to ICU survival status.

Variable	All Patients (n = 287)	Survivors (n = 169)	Non-Survivors (n = 118)	*p*-Value
Age, years	69 (62–75)	69 (62–74)	70 (61–75)	0.64
APACHE II score	20 (15–27)	17 (13–22)	26 (20–31)	<0.001
ICU length of stay, days	4 (2–9)	4 (2–6)	5 (1–15)	0.19
Male sex	173 (60.3)	101 (59.8)	72 (61.0)	0.90
Hematologic malignancy	41 (14.3)	15 (8.9)	26 (22.0)	0.002
Solid tumor	246 (85.7)	154 (91.1)	92 (78.0)	0.002
Advanced solid tumor	117 (47.6)	79 (51.3)	38 (41.3)	0.15
Metastatic solid tumor	38 (15.4)	14 (9.1)	24 (26.1)	<0.001
Positive blood culture	56 (19.5)	20 (11.8)	36 (30.5)	<0.001
Chemotherapy/immunotherapy	120 (41.8)	54 (32.0)	66 (55.9)	<0.001
Radiotherapy	54 (18.8)	21 (12.4)	33 (28.0)	0.001
Surgery performed	209 (72.8)	146 (86.4)	63 (53.4)	<0.001
ECOG 1	152 (53)	107 (63.3)	45 (38.1)	<0.001
ECOG 2	51 (17.8)	25 (14.8)	26 (22.0)	<0.001
ECOG 3	51 (17.8)	28 (16.6)	23 (19.5)	<0.001
ECOG 4	33 (11.5)	9 (5.3)	24 (20.3)	<0.001
Admission priority 1	232 (80.8)	156 (92.3)	76 (64.4)	<0.001
Admission priority 2	21 (7.3)	8 (4.7)	13 (11.0)	<0.001
Admission priority 3	2 (0.7)	0 (0.0)	2 (1.7)	<0.001
Admission priority 4	32 (11.1)	5 (3.0)	27 (22.9)	<0.001
Mechanical ventilation >48 h or death within 48 h while mechanically ventilated	135 (47.0)	39 (23.1)	96 (81.4)	<0.001
Vasopressor therapy >48 h or death within 48 h while receiving vasopressors	170 (59.2)	66 (39.1)	104 (88.1)	<0.001

Data are median (IQR) or n (%). Percentages for solid-tumor subcategories use patients with solid tumors as the denominator; other percentages use the column total. The *p*-value for ECOG status is based on an omnibus chi-square test and is repeated for each category row. The overall *p*-value for admission-priority categories was calculated using the Fisher–Freeman–Halton exact test because of sparse cell counts. APACHE II, Acute Physiology and Chronic Health Evaluation II; ECOG, Eastern Cooperative Oncology Group; ICU, intensive care unit.

**Table 2:** Univariable logistic regression for ICU mortality.

Variable	OR (95% CI)	*p*-Value
Female sex	0.95 (0.59–1.54)	0.83
Age, per year	1.00 (0.99–1.00)	0.56
ICU length of stay, per day	1.00 (0.99–1.00)	0.30
APACHE II score, per point	1.16 (1.12–1.21)	<0.001
Admission priority 4	9.73 (3.62–26.14)	<0.001
Hematologic malignancy	2.90 (1.46–5.76)	0.002
ECOG 4	6.16 (2.66–14.31)	<0.001
Mechanical ventilation >48 h or death within 48 h while mechanically ventilated	14.55 (8.10–26.12)	<0.001
Vasopressor therapy >48 h or death within 48 h while receiving vasopressors	11.59 (6.13–21.94)	<0.001
Advanced solid tumor	1.04 (0.53–2.06)	0.91
Lymph-node involvement	1.64 (0.82–3.27)	0.16
Metastasis	5.14 (2.33–11.33)	<0.001
Radiotherapy	2.76 (1.50–5.09)	0.001
Chemotherapy/immunotherapy	2.76 (1.69–4.51)	<0.001
Surgery	0.18 (0.10–0.32)	<0.001
Positive blood culture	3.15 (1.70–5.83)	<0.001
Positive urine culture	2.33 (1.15–4.76)	0.02

APACHE II, Acute Physiology and Chronic Health Evaluation II; CI, confidence interval; ECOG, Eastern Cooperative Oncology Group; ICU, intensive care unit; OR, odds ratio. Reference categories were priorities 1–3 for priority 4, solid malignancy for hematologic malignancy, ECOG 1 for ECOG 4, non-surgical admission for surgery, and absence of the respective exposure for binary variables.

**Table 3:** Multivariable logistic regression including mortality predictors available at ICU admission.

Variable	OR	95% CI	*p*-Value
Age, per year	0.99	0.96–1.01	0.286
APACHE II score, per point	1.15	1.10–1.21	<0.001
ECOG 2 vs. 1	2.14	0.95–4.83	0.066
ECOG 3 vs. 1	1.23	0.53–2.82	0.629
ECOG 4 vs. 1	6.37	2.05–19.78	0.001
Surgical/postoperative vs. non-surgical admission	0.29	0.14–0.60	0.001
Hematological vs. solid malignancy	1.02	0.38–2.76	0.963

Post-admission variables were excluded from this model. APACHE II, Acute Physiology and Chronic Health Evaluation II; CI, confidence interval; ECOG, Eastern Cooperative Oncology Group; ICU, intensive care unit; OR, odds ratio.

**Table 4:** Exploratory multivariable analysis of ICU-course characteristics associated with ICU mortality.

ICU-Course Characteristic	Adjusted OR	95% CI	*p*-Value
Mechanical ventilation >48 h or death within 48 h while mechanically ventilated	6.79	3.57–12.91	<0.001
Vasopressor therapy >48 h or death within 48 h while receiving vasopressors	4.33	2.11–8.92	<0.001
Positive blood culture	2.02	0.96–4.28	0.065

OR, odds ratio; CI, confidence interval. The organ-support variables are composite ICU-course measures incorporating either support lasting >48 h or death within 48 h while receiving the respective support. This exploratory analysis describes associations with ICU mortality and should not be interpreted as an analysis of the effect of organ-support duration.

## Data Availability

The data are available from the corresponding author upon reasonable request.
